# Genetic markers of olive fruit weight selected to be used in breeding experiments

**DOI:** 10.1007/s11032-025-01562-4

**Published:** 2025-04-07

**Authors:** Martín Moret, Alicia Serrano, Angjelina Belaj, Lorenzo León, Raúl de la Rosa, Francisco Luque

**Affiliations:** 1 Departamento de Biología Experimental, Instituto Universitario de Investigación en Olivar y Aceites de Oliva, Universidad de Jaén, 23071 Jaén, Spain; 2 Centro de Investigación y Formación Agraria de Alameda del Obispo, Instituto de Investigación y Formación Agraria y Pesquera (IFAPA), 14004 Córdoba, Spain; 3 Instituto de Agricultura Sostenible (IAS), CSIC, 14004 Córdoba, Spain

**Keywords:** *Olea europaea*, Genetic markers, Fruit weight, Breeding

## Abstract

Olive fruit weight is a crucial trait to consider in olive breeding programs due to its impact on final yield and its relevance for mechanical harvesting and fruit processing. Although environmental conditions influence this trait, fruit weight is primarily determined by genetic factors and exhibits a high degree of heritability in breeding progenies. Despite several studies identifying potential markers associated with fruit weight, these markers have not been validated. In this study, we analyzed 40 genetic markers linked to fruit weight using a dataset comprising 73 cultivars (including 33 newly sequenced varieties) and 10 wild olives with a wide range of phenotypic characteristics, spanning from very light (0.41 g) to very heavy fruits (8.57 g). By examining the phenotype distribution for each genotype of the newly sequenced varieties, we successfully validated 16 genetic markers. Additionally, machine learning tools demonstrated that 9 out of the 16 validated markers have a high predictive ability for fruit weight. As a result, our work provides, for the first time, a set of 9 well-validated genetic markers suitable for use in marker-assisted selection during the early stages of olive breeding programs.

## Introduction

Assessing fruit weight is a critical trait when evaluating and selecting olive varieties in breeding programs (Fernández-Escobar et al. 2013). Fruit weight is a critical component of final yield, along with fruit number and oil content, making it an essential factor in variety selection and improvement efforts (De la Rosa et al. 2008). Additionally, it significantly impacts suitability for mechanical harvesting, particularly by trunk shakers in vase-formed trees (Lavee et al. 1982). As the olive industry continues to evolve, understanding and optimizing fruit weight becomes increasingly important to ensure the sustainability and efficiency of olive production systems (Fernández-Escobar et al. 2013). This will contribute to developing more efficient and environmentally friendly varieties and allow farmers to improve the quality and quantity of their harvests. Both the environment and genotype have demonstrated a significant influence on the fruit weight (Zeinanloo et al. 2009; León et al. 2016; Mousavi et al. 2019). Among the environmental factors, water availability appears to be the most important one (Conde-Innamorato et al. 2022; Sánchez-Piñero et al. 2024), followed by air temperature (Miserere et al. 2023). Fruit yield could also significantly impact fruit weight (Rosati et al. 2010; Fernández et al. 2018). On the other hand, high genetic variability has been widely reported for fruit weight in many variety evaluations (Linos et al. 2014). This trait has also received considerable attention in breeding programs, both for oil (Yılmaz-Düzyaman et al. 2023) and table purposes (Morales-Sillero et al. 2011). In fact, it has shown a high degree of genetic influence and heritability in breeding progenies (Arias-Calderón et al. 2014) and variety trials conducted in different environments (Mousavi et al. 2019).

Several studies have reported a wide range of genes implicated in fruit development, including cell production in apples (Dash and Malladi 2012) and cherries (De Franceschi et al. 2013). In olives, differences in fruit weight among varieties are related to cell number rather than cell size (Rosati et al. 2010, 2020). Cell division in fruit occurs only a few weeks after flower anthesis (Camarero et al. 2023). In fact, some RNAs putatively involved in olive fruit development show high induction right after fruit set (Serrano et al. 2024). However, little is known about the genetic basis of fruit weight variability. Only a few markers associated with fruit weight have been found in an ‘Arbequina’ x ‘Picual’ linkage map (Atienza et al. 2014), in a genome-wide association study (GWAS) together with an olive biodiversity study (Bazakos et al. 2023) and another one performed with Genotyping by Sequencing (GBS) instead of whole genome sequences (Kaya et al. 2019). However, none of these studies included a validation of the genetic markers (GMs). A recent GWAS study from our group found a series of probable GMs to determine fruit weight (Moret et al. 2023). This study is the only one with a validation of the GMs. However, that validation was performed with a ‘Frantoio’ x ‘Picual’ segregating progeny and some markers could not be tested because both varieties were homozygous for one allele of the GM, while for other GMs a homozygote was missing in the progeny. This fact was limiting for the validation of the GMs. Given the long juvenile period in olive trees (Santos-Antunes et al. 2005), it would be of great interest for breeding programs to have GMs that could estimate fruit weight phenotype at the seedling stage.

GWAS is a powerful tool to uncover the genetic basis of complex traits in plants (Korte and Farlow 2013). This methodology allows to analyze natural genetic variation in diverse populations, identifying associations between single nucleotide polymorphisms (SNPs) and phenotypes of interest (Huang and Han 2014). Regarding olive, GWAS has proven to be particularly valuable due to the genetic complexity and long history of domestication of this species (Besnard et al. 2018). The application of GWAS in olive can not only facilitate the understanding of the genetic architecture of important traits such as fruit weight, but can also provide potential markers for marker-assisted selection (MAS), thus accelerating breeding programs (Kaya et al. 2019).

However, GWAS also has limitations in confirming the identified GMs. False positive associations may arise due to population structure, relatedness among individuals, and multiple testing issues (Korte and Farlow 2013). Furthermore, GWAS may fail to detect rare variants or those with small effect sizes, leading to lack of heritability (Tam et al. 2019). To overcome these limitations and confirm the validity of GMs, several strategies are employed. Moreover, functional validation through molecular biology techniques, such as gene expression analysis, genetic transformation, and gene editing, is necessary to establish the causal relationship between the identified markers and the trait of interest (Huang and Han 2014). However, these methods of confirmation do not really validate the predictability of the GMs.

Machine learning techniques have gained prominence in identifying important GMs associated with complex traits in ecology and evolution, including plant genetics (Brieuc et al. 2018). Among these techniques, Random Forest (RF) has emerged as a powerful tool for analyzing genomic data and uncovering gene-trait associations. RF is an ensemble learning method that builds multiple decision trees and combines their predictions to improve accuracy and reduce overfitting (Breiman 2001). This algorithm can efficiently analyze thousands of loci simultaneously and account for non-additive interactions, making it particularly useful for genomic studies where the number of GMs often far exceeds the number of samples (Brieuc et al. 2018). Random Forests are used for both classification and regression tasks, and the final prediction is determined by aggregating the outputs of all trees (Louppe et al. 2013). Additionally, RF provides measures of variable importance, allowing researchers to identify the most influential GMs for the trait of interest (Louppe et al. 2013).

One of the key advantages of RF over other machine learning techniques, such as neural networks, is its ability to rank the importance of input features, such as GMs, based on their contribution to the predictive performance of the model (Louppe et al. 2013). This ranking of feature importance is achieved through a process called"permutation importance"or"mean decrease in accuracy,"where the impact of each feature on the model accuracy is assessed by randomly permuting its values (Breiman 2001). Unlike neural networks, which often act as"black boxes"with limited interpretability, RF provides clear insights into which features (in our case, GMs) are most influential in determining the trait of interest. Additionally, RF is less prone to overfitting compared to many other algorithms, particularly when dealing with high-dimensional data typical of genomic studies (Brieuc et al. 2018). It can handle both linear and non-linear relationships between features and the target variable, making it suitable for capturing complex genetic interactions. Furthermore, RF is relatively robust to outliers and noise in the data, a common challenge in biological datasets (Chen and Ishwaran 2012). These characteristics make RF an excellent choice for analyzing GMs and their association with traits like fruit weight in olive varieties, complementing the findings from GWAS studies and potentially uncovering additional important genetic factors.

In a previous GWAS study conducted by Moret et al., (2023), 38 clusters of GMs associated with the fruit weight in olive were identified. While some of these GMs could be validated using a ‘Frantoio’ x ‘Picual’ progeny, validation was impossible for many others. To address this limitation, in the present study new olive varieties that were not included in the original GWAS were sequenced, and these additional samples were used to validate the GMs. The main objective of this work was to pinpoint a reduced set of GMs that account for the majority of the variation in fruit weight. To achieve this goal, 33 new olive varieties were sequenced and used to validate the markers obtained in the previous GWAS. Additionally, we wanted to determine if it was possible to apply Random Forest to validate the GMs and obtain a smaller subset to be used in breeding programs.

## Material and methods

### Plant material and fruit weight determination

A total of 73 varieties of the World Olive Germplasm Bank of Córdoba IFAPA (WOGBC) (Belaj et al. 2022) were selected for maximizing the variability for fruit weight and genetic variability (Belaj et al. 2018, 2012 and WOGBC unpublished data). Additionally, a set of 10 wild olives coming available from previous prospecting surveys were also selected (Belaj et al. 2011). This material included the 40 varieties and 10 wild olives included in a previous GWAS study (Moret et al. 2023) and 33 new varieties for this study. Trees of all selected genotypes, and having sufficient fruit load, were sampled (1 kg of olives) during two harvesting seasons. Three subsamples of around 25 g were randomly selected to measure fruit fresh weight.

### Next generation sequencing

Fresh leaves of 33 new WOGBC varieties were sampled to extract DNA using the Cytiva DNA Extraction Kit PhytoPure (Global Life Science Solutions Operations UK Ltd., Buckinghamshire, UK). Subsequently, the DNA samples were sequenced using the Illumina NovaSeq 6000 System at Novogene (Novogene Co., Ltd., Cambridge, UK). Each DNA sample yielded at least 70 gigabases of paired-end sequences with a read length of 150 × 2 bases. The sequenced genomes were aligned to the reference genome “Picual” Oleur061 (Jiménez-Ruiz et al. 2020) using the command line program bowtie2, which is specifically designed for small- to medium-sized sequences (Langmead and Salzberg 2012). The alignment files were merged using bamaddrg, and Variant Call Format (VCF) files containing genetic variants were obtained using freebayes (v1.3.6).

### Statistical analysis of the genetic markers in new varieties

GM confirmation was made by comparing the fruit weight distribution among the three possible genotypes. First, normal distribution was verified using the Kolmogorov–Smirnov test. Then, a t-student test was performed to determine if there were differences in fruit weight among the three genotypes of each GM, heterozygous and both types of homozygous. A significance threshold of 0.05 was applied in the t-test.

### Machine learning analysis of predictive genetic markers

Genotypic and phenotypic data from the entire set of sequenced varieties, 33 in this work and 50 in (Jiménez-Ruiz et al. (2020), were used to determine the predictive value of GMs. Using the information from the total sequenced varieties, a random forest model was trained using the Python scikit-learn libraries. During preprocessing, missing data were imputed using the mean value, and hyperparameter optimization for model training was performed using GridSearchCV, applying cross-validation for reliability improvement of the results.

Once the model was trained, an analysis of the importance of the features (in our case, the GMs) was performed to find out which ones had the greatest impact on the model. These GMs with the greatest impact on the model will be those that show the greatest impact on the target variable (fruit weight). By identifying them, we can corroborate the results obtained from classical statistical tests and determine if there is a smaller group that significantly influences fruit weight. To evaluate the predictive performance of the model, we calculate the mean square error (MSE), the mean absolute error (MAE) and coefficient of determination (R^2^) by comparing the actual values with the predictions made by the model on the test set.

## Results

### Newly sequenced varieties

**Fig. 1:**
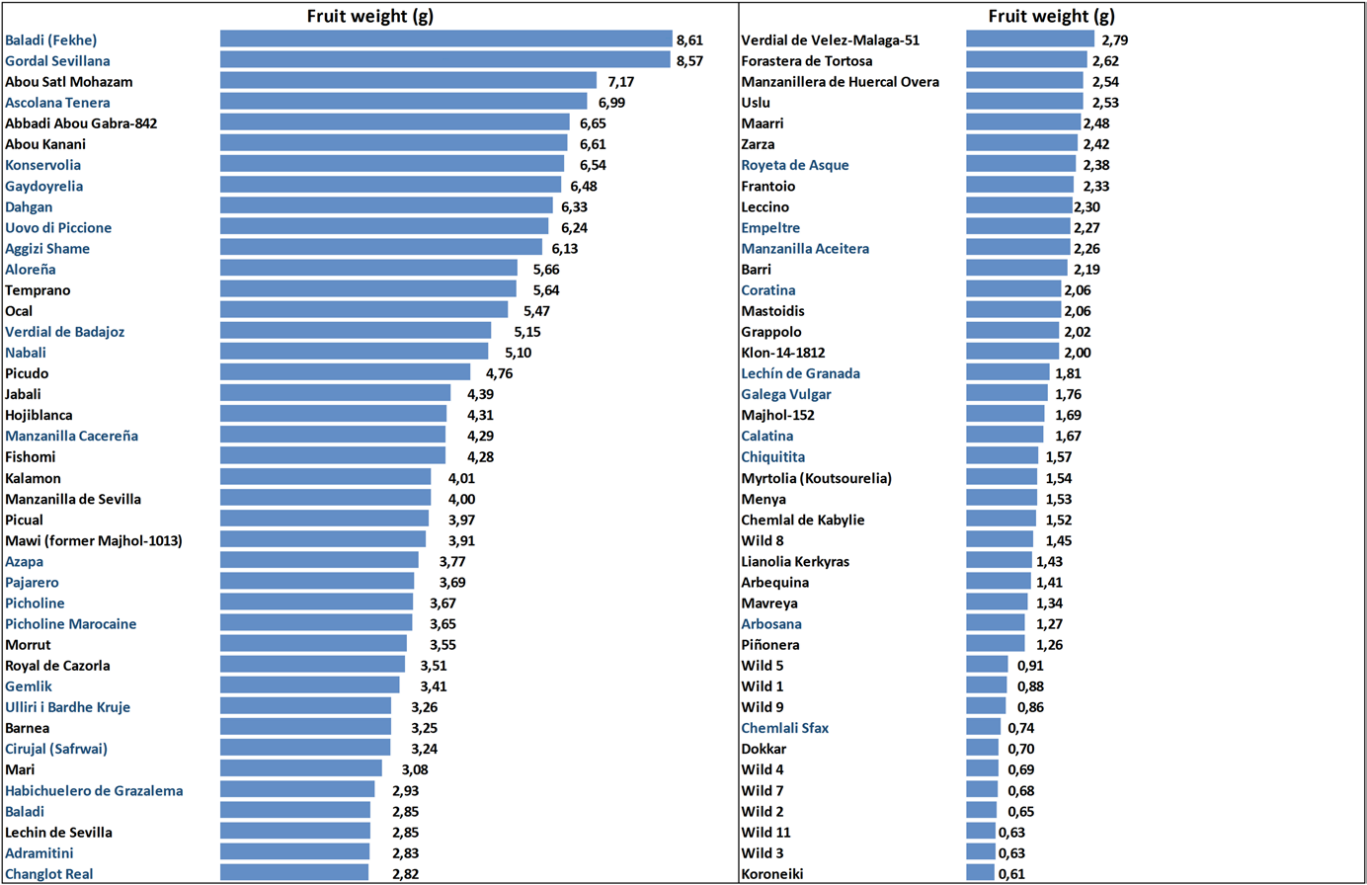
Fruit weight of the sequenced varieties. Varieties in blue were sequenced in this work and in black were sequenced by Jiménez-Ruiz et al. 2020

To validate the GMs previously identified through genome-wide association studies (GWAS) by Moret et al. (2023), we sequenced 33 new varieties. Figure 1 presents the fresh fruit weight data of a list of these 33 newly sequenced varieties, along with 40 other varieties and 10 wild accessions that were previously sequenced by Jiménez-Ruiz et al. (2020). The weight data was obtained from former (Del Rio et al. 2005, Belaj et al. 2012) and ongoing studies conducted at the WOGBC, Spain. A VCF file containing the 83 genotypes was obtained for use in the GMs validation analysis.

### Validation of genetic markers

The GMs previously identified through GWAS by Moret et al., (2023), that consisted in SNPs variations, had been initially grouped into 38 clusters based on their proximity in the scaffold and their co-segregation. In the current study, we reorganized them into 40 clusters, splitting two of them due to variants that seemed to correspond to different chromosomes in the Oe450 genome (Unver et al. 2017). For this study, only one GM from each cluster was considered. The ability of those GM for explaining the variability of fruit weight data was evaluated in the 33 newly sequenced varieties. Since these varieties were not part of the GWAS, they served as a confirmatory set of samples.

Using a significance level of 0.05 in the t-test, 16 GMs, of the 40 considered, were confirmed as a consistent predictors for fruit weight in the 33 newly sequenced varieties. Importantly, this result did not conflict with the previous segregating progeny validation. Other 19 GMs did not meet this criterion and were therefore not considered as validated predictors. And the remaining 5 GMs could not be analyzed due to the absence of their sequences in a large number of varieties (Table 1).

**Table 1:** Confirmatory analysis of genetic markers in the 33 newly sequenced varieties

Scaffold	GM Position in Scaffold	Alleles	Previous GM confirmation (Moret et al. 2023)	GM analysis in 33 new genomes (This study)			
				GM confirmation	*P* value 0/0 vs 0/1	*P* value 0/0 vs 1/1	*P* value 0/1 vs 1/1
**Oleur061Scf0014**	**300237**	**A/G**	**-**	**YES**	** < 0.05**	** < 0.05**	**0.35**
Oleur061Scf0029	478377	G/T	-	NO	0.34	0.76	0.32
Oleur061Scf0029	881555	G/A	NO	NO	0.12	ND	ND
Oleur061Scf0029	890780	A/G	NO	YES	0.48	< 0.05	0.06
Oleur061Scf0091	468627	A/G	YES	NO	0.43	0.26	0.61
Oleur061Scf0091	491341	G/A	YES	NO	0.52	0.45	0.74
Oleur061Scf0122	385110	C/T	-	NO	0.99	ND	ND
Oleur061Scf0148	495009	C/T	NO	NO	0.19	ND	ND
Oleur061Scf0193	1194745	A/G	NO	YES	< 0.05	ND	ND
**Oleur061Scf0306**	**643067**	**C/T**	**YES**	**YES**	** < 0.01**	** < 0.00001**	** < 0.01**
Oleur061Scf0340	201854	ATT/GTC	-	-	-	-	-
Oleur061Scf0360	830983	A/T	-	NO	ND	0.31	ND
**Oleur061Scf0476**	**81329**	**G/A**	**-**	**YES**	** < 0.05**	**ND**	**ND**
Oleur061Scf0503	499553	GTT/CTC	-	NO	ND	0.26	ND
Oleur061Scf0871	153993	C/G	-	-	-	-	-
**Oleur061Scf0960**	**26182**	**A/G**	**-**	**YES**	**0.27**	** < 0.05**	**0.09**
**Oleur061Scf1178**	**622380**	**G/T**	**-**	**YES**	**0.79**	**0.06**	** < 0.01**
**Oleur061Scf1459**	**972705**	**C/A**	**-**	**YES**	** < 0.05**	**ND**	**ND**
**Oleur061Scf1787**	**58484**	**A/C**	**-**	**YES**	**0.49**	** < 0.05**	**0.17**
**Oleur061Scf2091**	**176408**	**A/C**	**-**	**YES**	** < 0.05**	** < 0.01**	**0.09**
Oleur061Scf2874	871356	A/G	YES	NO	0.74	0.60	0.42
**Oleur061Scf3270**	**126286**	**TC/AA**	**-**	**YES**	** < 0.05**	** < 0.01**	**0.38**
**Oleur061Scf3346**	**640676**	**G/A**	**YES**	**YES**	** < 0.01**	** < 0.000001**	** < 0.001**
Oleur061Scf3361	582298	A/C	YES	NO	0.20	ND	ND
**Oleur061Scf3663**	**386986**	**G/A**	**YES**	**YES**	** < 0.01**	** < 0.05**	**0.23**
Oleur061Scf3825	171174	T/C	-	NO	0.79	0.94	0.73
**Oleur061Scf4112**	**400456**	**C/A**	**-**	**YES**	** < 0.05**	**ND**	**ND**
Oleur061Scf4112	422150	C/G	NO	NO	0.12	ND	ND
Oleur061Scf4351	26325	G/C	-	NO	0.61	0.70	0.83
Oleur061Scf4403	193259	T/C	NO	NO	0.08	ND	ND
Oleur061Scf4462	60219	T/C	-	-	-	-	-
Oleur061Scf4491	299627	C/G	-	NO	ND	0.52	ND
**Oleur061Scf4878**	**118649**	**C/G**	**YES**	**YES**	** < 0.05**	** < 0.01**	** < 0.01**
Oleur061Scf4977	92722	T/G	-	-	-	-	-
Oleur061Scf5420	23710	C/T	NO	YES	< 0.05	0.07	0.91
Oleur061Scf5641	84939	T/C	YES	NO	0.47	ND	ND
**Oleur061Scf6972**	**73917**	**A/G**	**YES**	**YES**	**0.05**	**0.61**	**0.64**
Oleur061Scf7206	20070	CACG/CGCA	-	-	-	-	-
**Oleur061Scf7731**	**48236**	**G/A**	**-**	**YES**	**0.38**	** < 0.01**	** < 0.01**
**Oleur061Scf8230**	**14454**	**T/C**	**-**	**YES**	**0.86**	**0.06**	** < 0.01**

(0/0) homozygous genotype for the reference variant allele (the first in the Allele column).

(0/1) heterozygous genotype.

(1/1) homozygous genotype for the alternative variant allele (the second in the Allele column).

ND = not determined because insufficient data.

If any of the comparisons 0/0 vs 0/1, 0/0 vs 1/1 or 0/1 vs 1/1 had a p-value lower than 0.05 the GM was confirmed.

The 16 confirmed GMs are printed in bold type.

**Fig. 2:**
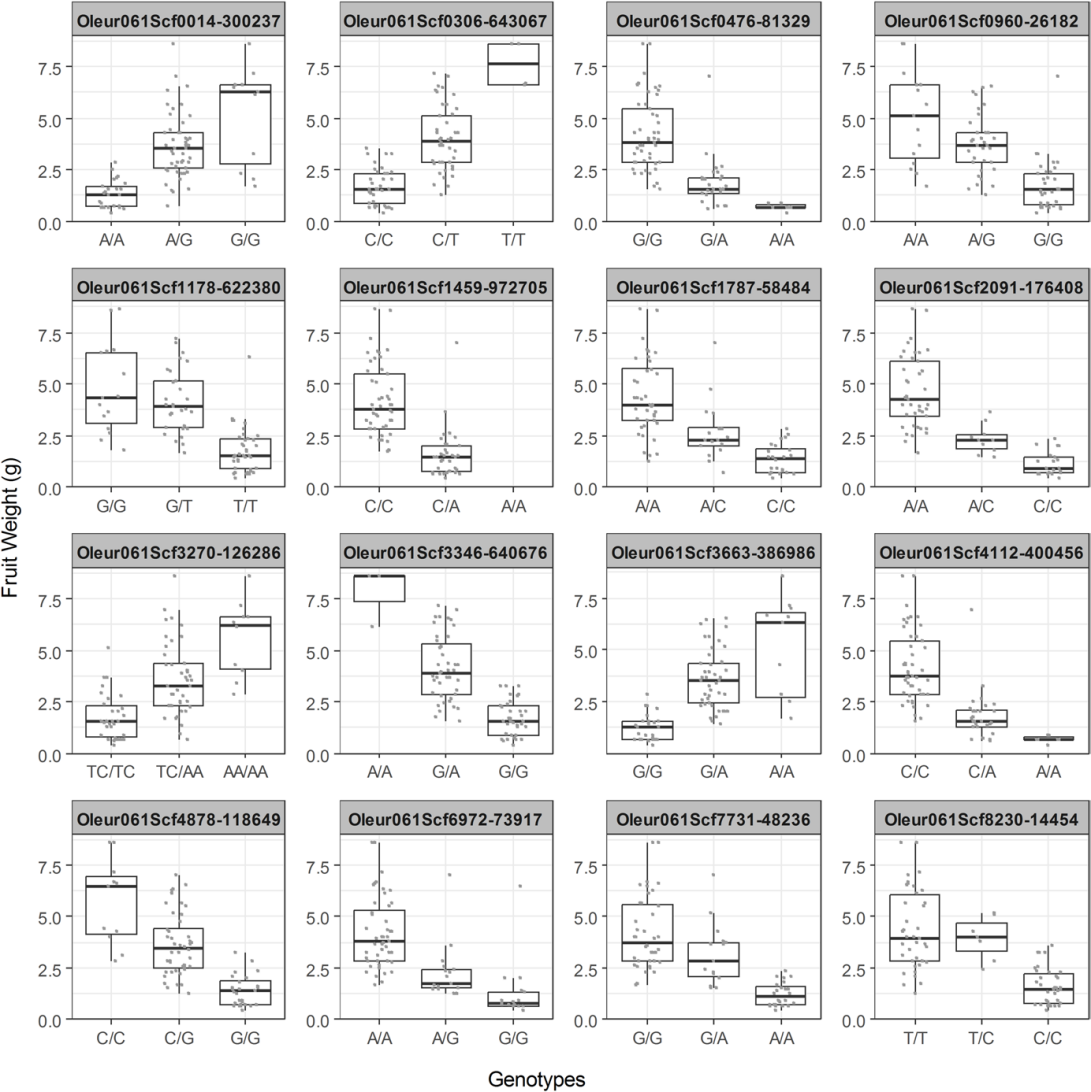
Boxplots of confirmed GMs. The 16 confirmed GM are represented. For each GM, the phenotypic distribution found in 73 sequenced varieties and 10 wild olive trees of the three possible genotypes is represented. The horizontal bar in each box represents the median of the phenotype distribution

The whole set of 83 genotypes, the 50 included in the GWAS study (Moret et al. 2023) and the 33 newly sequenced in this study were used to represent the phenotypic distribution for each confirmed GM (Fig. 2).

### Genetic markers prediction model based on machine learning

Once it was determined that there were a total of 16 validated GMs that affected fruit weight determination, we applied Random Forests to corroborate these results and identify a smaller subset of GMs that account for the majority of the variation in fruit weight. Prior to this analysis, the Oleur061Scf1459 - 972705 GM was excluded from the study. This particular GM is located in the OeMed15a gene, which codes for a component of the Mediator complex, a coactivator involved in transcriptional activation. Notably, the essential role of this Mediator complex and the absence of homozygous varieties for the “A” allele may suggest that this gene could be a lethal one.

**Fig. 3:**
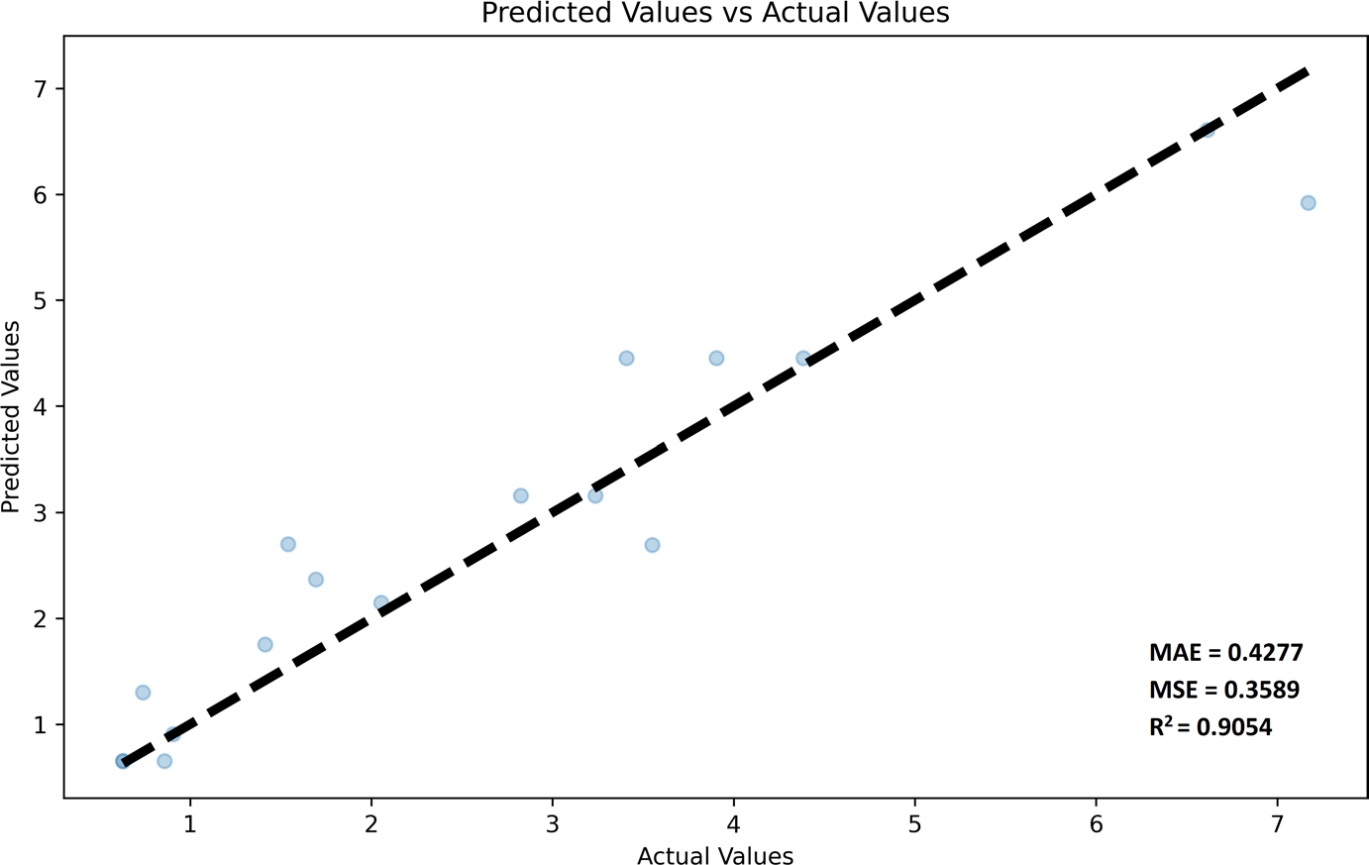
Predictive ability of the model. The model’s predictive ability is assessed by adjusting it to the test varieties. The Mean Absolute Error (MAE), Mean Square Error (MSE) and Determination Coefficient (R^2^) are provided. Predicted and actual fruit weight are represented in grams

After performing a Random Forest Regression analysis, nine of the 15 GMs considered were identified as having significant relevance in predicting fruit weight. These individual GMs had significances ranging from 5.20% to 13.65% (Table 2). The importance of each GM was determined through a model feature significance analysis, taking into account the contribution of the GM to impurity reduction in the forest decision trees. When considered together, these GMs contribute significantly to the predictive capability of the model. Consequently, they were selected to evaluate their suitability as predictors for fruit weight in new genotypes. Subsequent Random Forest Regression analysis confirmed that these GMs serve as strong predictors for fruit weight in the test varieties. This conclusion is supported by quality metrics, including an R-squared value of 0.9054, a mean absolute error (MAE) of 0.4277, and a mean squared error (MSE) of 0.3589 (Fig. 3).

**Table 2:** Random forest regression analysis of validated GMs

GM	Chromosome*	Relevance in the predictive model
**Oleur061Scf1787 - 58484**	**21**	**0.1365**
**Oleur061Scf0306 - 643067**	**18**	**0.1274**
**Oleur061Scf3346 - 640676**	**8**	**0.1129**
**Oleur061Scf6972 - 73917**	**21**	**0.0958**
**Oleur061Scf0476 - 81329**	**US**	**0.0856**
**Oleur061Scf0014 - 300237**	**18**	**0.0825**
**Oleur061Scf4112 - 400456**	**13**	**0.0780**
**Oleur061Scf3663 - 386986**	**8**	**0.0562**
**Oleur061Scf2091 - 176408**	**US**	**0.0520**
Oleur061Scf1178 - 622380	5	0.0477
Oleur061Scf7731 - 48236	8	0.0387
Oleur061Scf0960 - 26182	US	0.0338
Oleur061Scf3270 - 126286	7	0.0294
Oleur061Scf4878 - 118649	4	0.0166
Oleur061Scf8230 - 14454	15	0.0068

A GM lacking the alternative homozygous variant was removed from the analysis. The GMs finally selected are in bold. All together explain more than 80% of the fruit weight predictive model. *Olea europaea var. sylvestris genome was used as reference to determine the chromosomal location (Unver et al. 2017). US = unfound sequence in the wild genome.

**Fig. 4:**
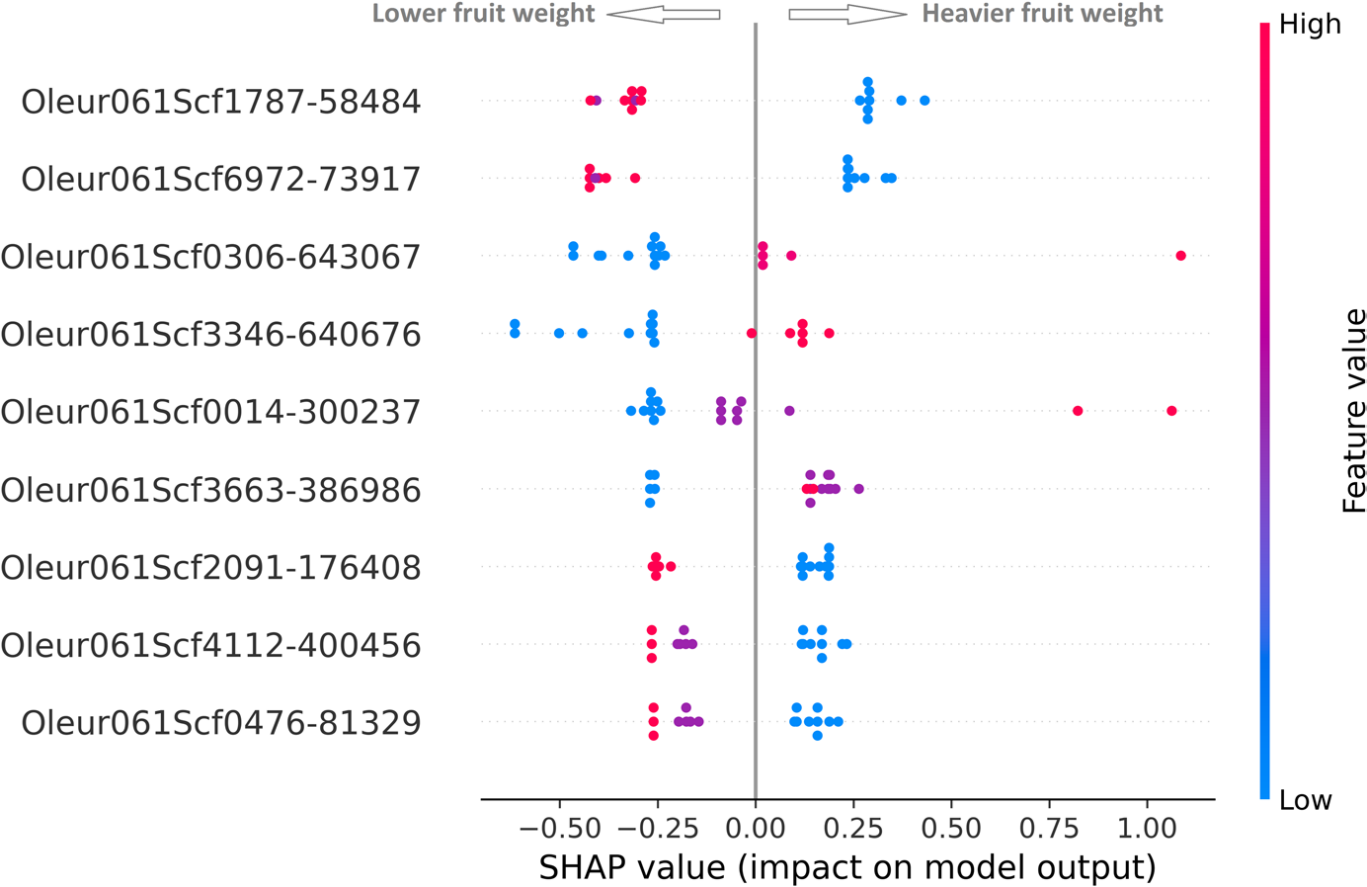
SHAP value of the 9 GMs selected as predictors of fruit weight in olive tree breeding. The blue symbols represent reference homozygous varieties, the purple symbols represent heterozygous varieties, and the red symbols represent alternative homozygous varieties

The final objective was to obtain a reduced set of GMs that contribute to the increase in fruit weight. To achieve this, an additional analysis was performed to determine the SHAP values (Fig. 4). The SHAP value for a specific GM represents the average marginal contribution of that marker across all possible combinations of the other markers in the model. In other words, it quantifies how much the presence or absence of a particular GM affects the model’s prediction, considering its interactions with other GMs. Positive SHAP values indicate that the presence of a GM contributes to an increase in the predicted fruit weight, while negative values suggest a decrease. By analyzing the SHAP values, we can identify which GMs are most influential in determining higher fruit weight, providing valuable insights for marker-assisted selection in breeding programs.

## Discussion

Olive fruit weight is a complex polygenic trait influenced by the interaction of the plant genotype and the environment. Nevertheless, the high genetic influence of this trait has been evidenced (Mousavi et al. 2019; Arias-Calderón et al. 2014). Therefore, the identification of GMs associated with this trait has become a highly desirable task.

Reporting GWAS studies and putative GMs without confirmatory experiments has limited utility. In a previous study, we conducted a GWAS analysis that identified 113 putatively GMs associated with fruit weight (Moret et al. 2023). These GMs were reorganized into 40 clusters. Subsequently, some of these GMs were examined in a “Frantoio x Picual” progeny. While a subset of them was confirmed as predictors for fruit weight, the parents and progeny exhibited a narrow distribution of fruit weight. Consequently, a significance level of 0.1 was stablished. Additionally, several GMs could not be analyzed due to homozygosity in both parents of the progeny. Therefore, it is of special relevance not only to confirm the GMs obtained and get a reduced subset of them, but also to find new methodologies that can reduce costs and help to simplify the current processes of GWAS marker confirmation.

Regarding the Random Forest results, the model presents good quality metrics, correctly approximating the model predictions to the real values of the test set. The values of the trait significance analysis showed that there are 9 GMs that contribute most notably to fruit weight, highlighting the markers Oleur061Scf1787 - 58484, Oleur061Scf0306 - 643067, Oleur061Scf3346 - 640676 and Oleur061Scf6972 - 73917 for jointly contributing to the 50% reduction of the model impurity, identifying through SHAP values the best haplotypes to increase fruit weight.

The heterozygous varieties for a specific GM may exhibit intermediate fruit weight, falling between those of the two homozygous genotypes. For example, this occurs in the case of the Oleur061Scf0014 - 300237 GM. Alternatively, some heterozygous varieties may have fruit weights quite similar to one of the homozygous genotypes, as seen in Oleur061Scf1787 - 58484 GM. It is interesting to note the consistency of phenotype distribution observed across the entire set of 83 genotypes (Fig. 2) and in the smaller set of test varieties, the SHAP value impacts on model output (Fig. 4).

The relative fraction of the phenotype explained by each GM (Table 2), should be considered when using this set of GMs as fruit weight predictors in breeding experiments. In addition to these 9 GMs, Oleur061Scf1459 - 972,705 GM should also be considered because the “A” allele might be a lethal gene, and heterozygotes produce smaller fruits (Fig. 2). Therefore, if medium or high-weight fruits are desired, breeding selection should include only genotypes with C/C for this particular GM.

The set of those 9 GMs that seems to be a good predictor of fruit weight variability, could be of great interest to improve the efficiency of olive breeding programs. In fact, low fruit weight seedlings are discarded from the first stage of breeding selection (León et al. 2015). The high percentage of seedlings with low fruit weight found in many breeding progenies (Yılmaz-Düzyaman et al. 2023) makes very desirable to have a consistent marker assisted selection system to discard them as early as possible. The consistency demonstrated by set of thee 9 GMs in identifying low fruit weight could be of great value to set up this system.

## Conclusions

In this study, a set of 9 GMs has been identified as excellent predictors of fruit weight in the tested olive varieties and the impact and role of each genotype has been described. The results demonstrate that the Random Forest algorithm can serve as an effective model for validating GMs obtained through a GWAS, enabling the acquisition of a reduced set of markers that significantly influence an agronomic quality trait, such as fruit weight determination. Consequently, these GMs are well-suited for use in a marker-assisted selection system in early stages of olive breeding programs, where fruit weight is one of the key traits of selection.

## Data Availability

Sequencing raw data is available at NCBI BioProject ID: PRJNA1124876.
